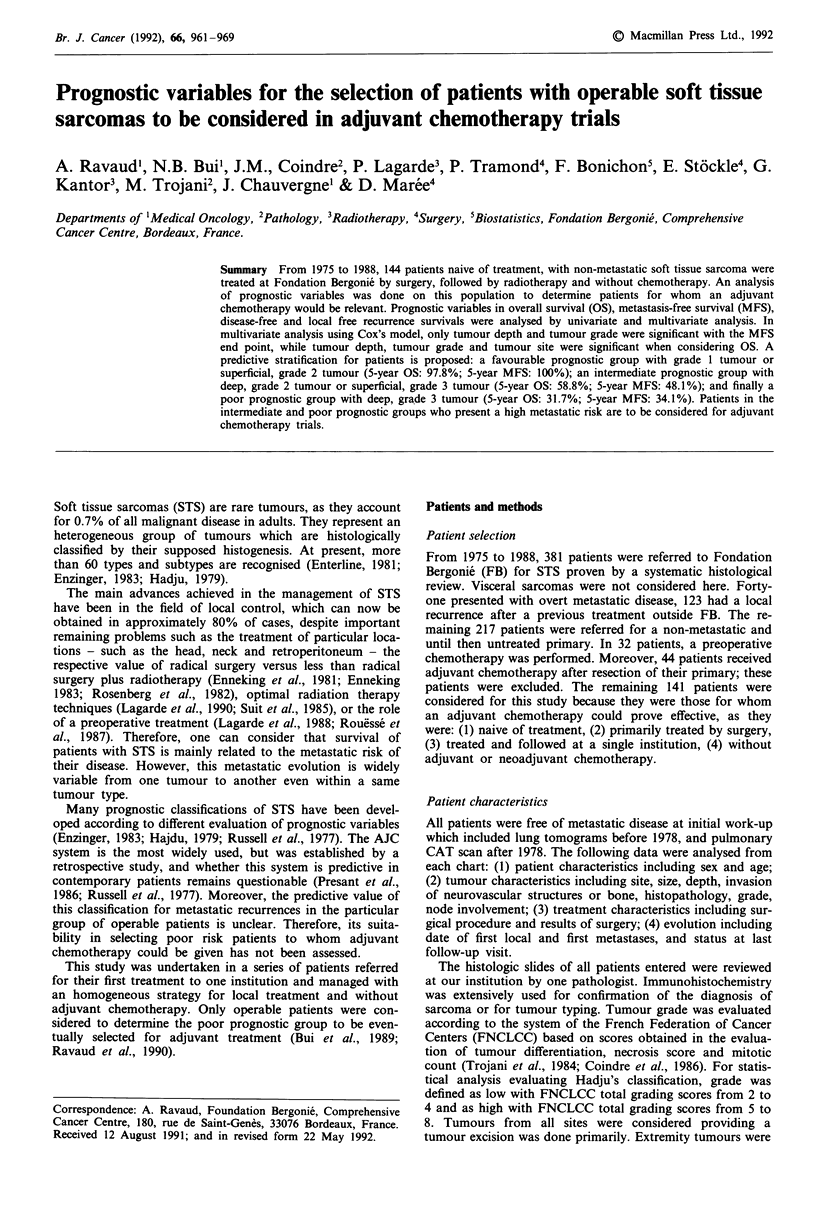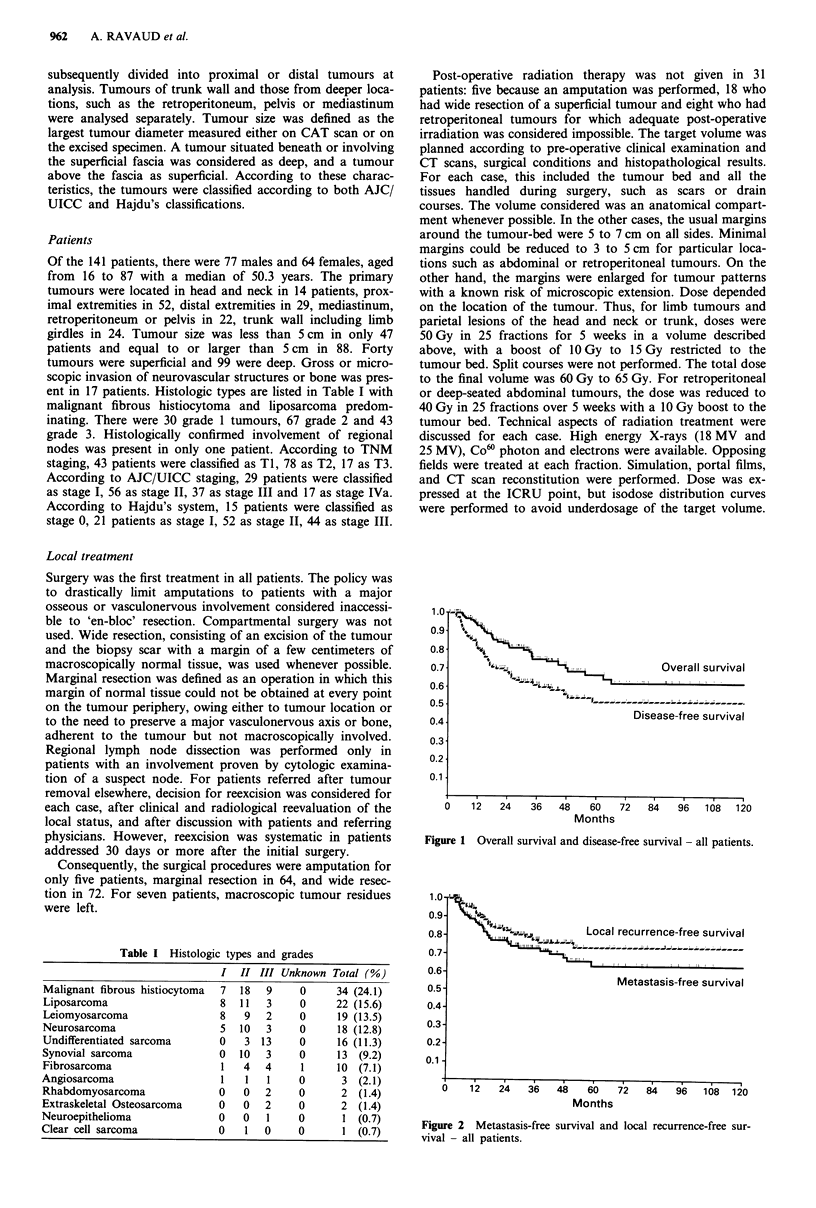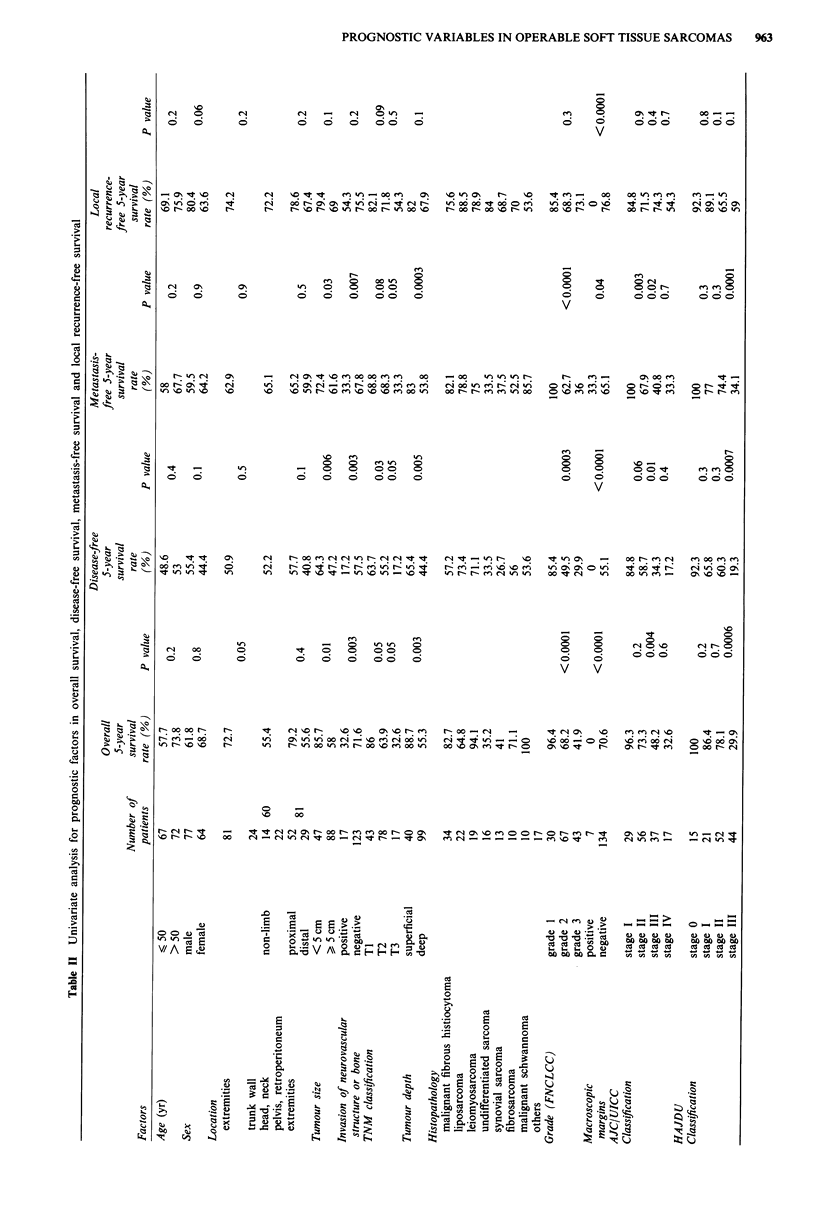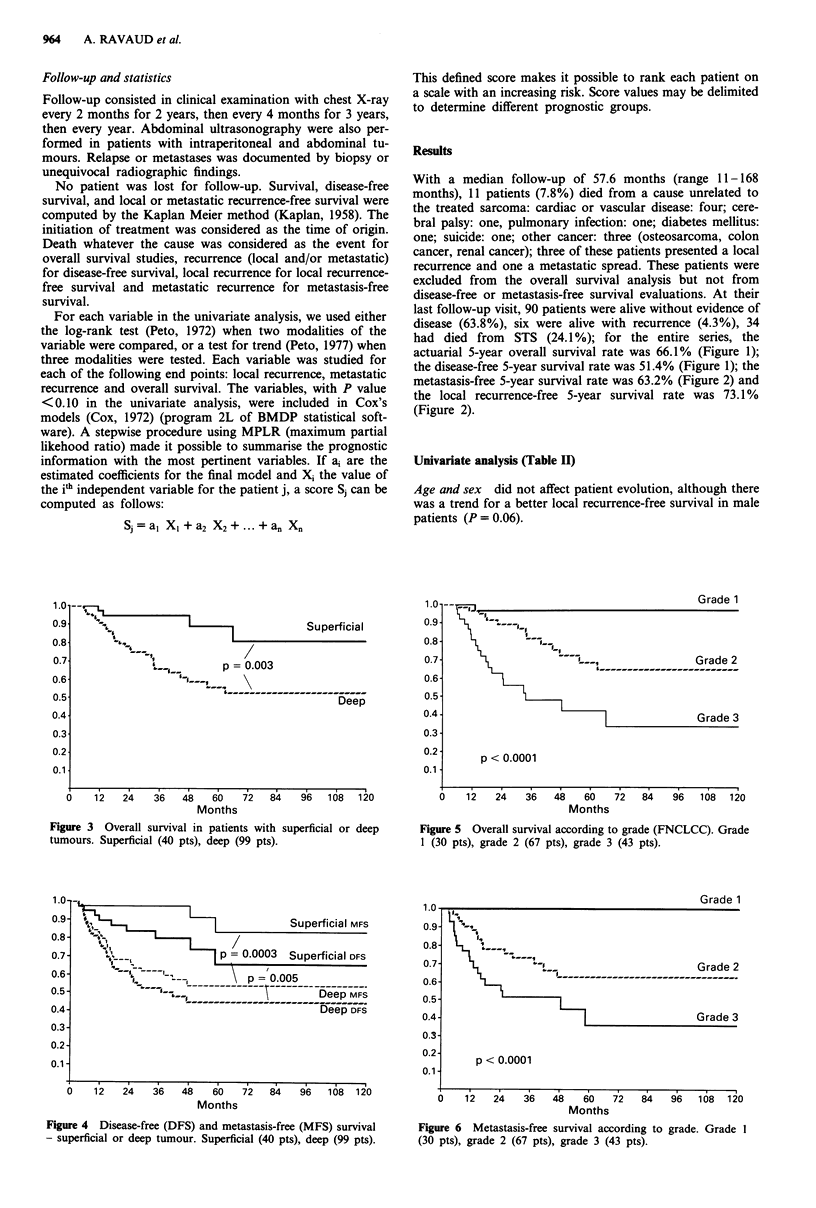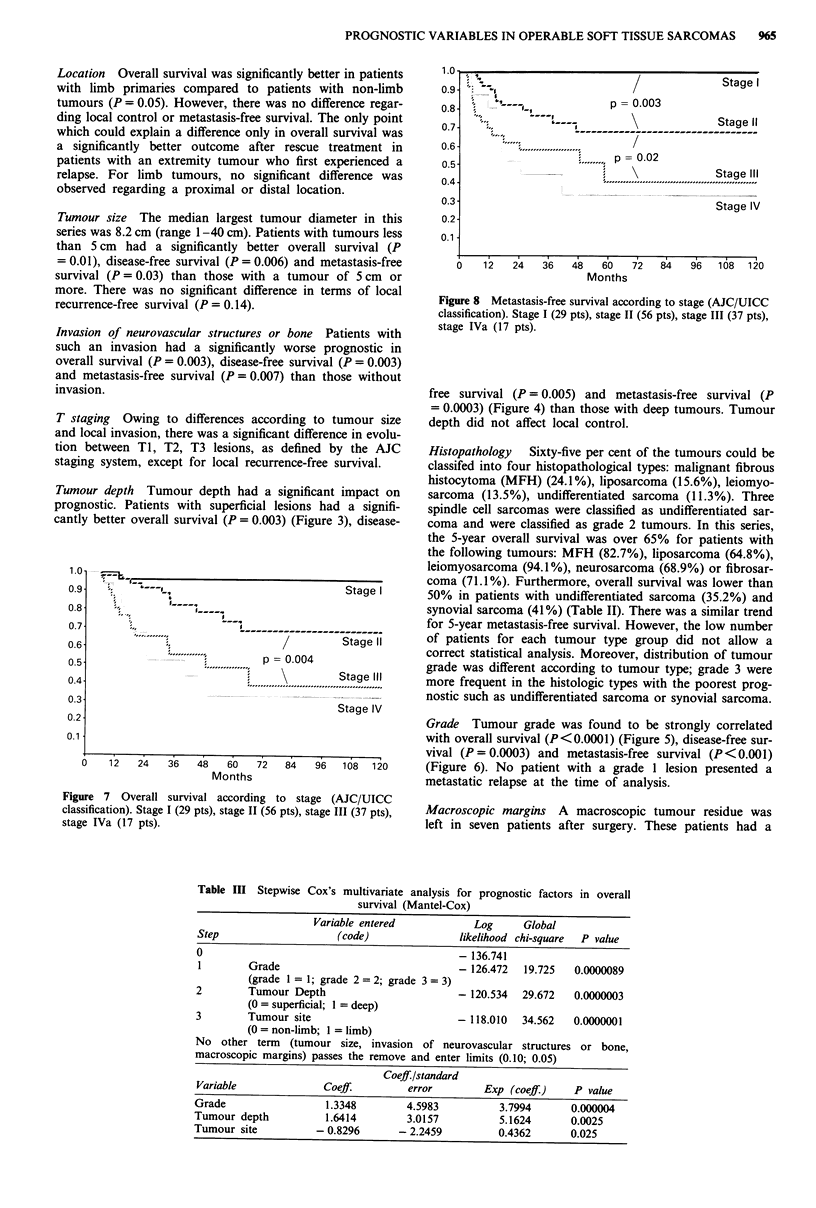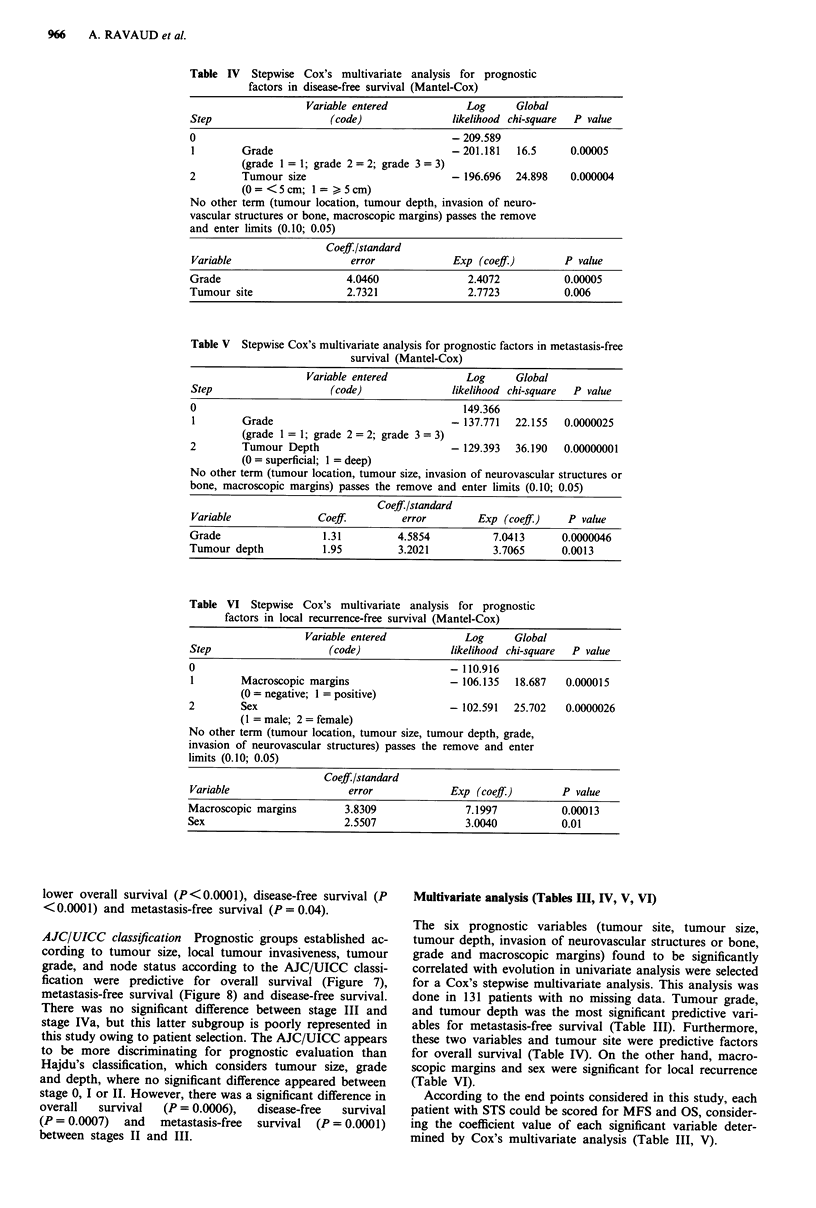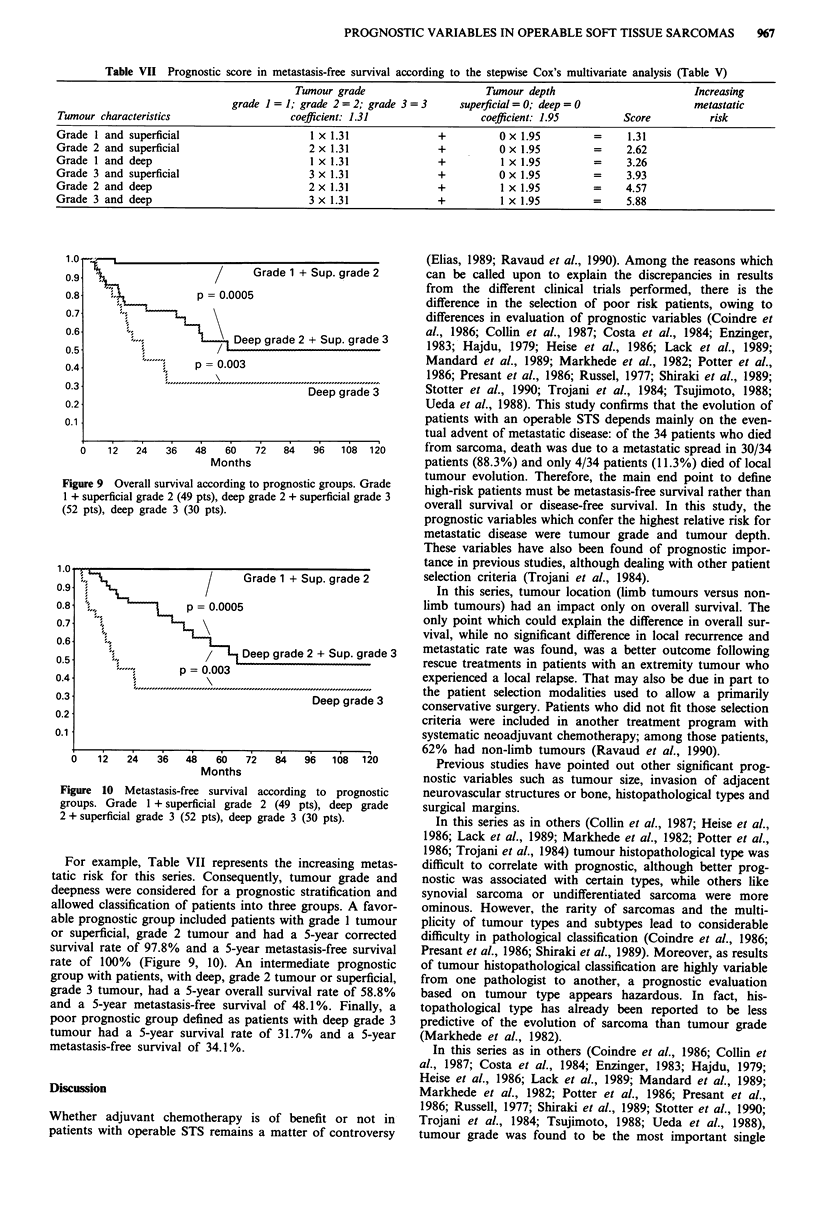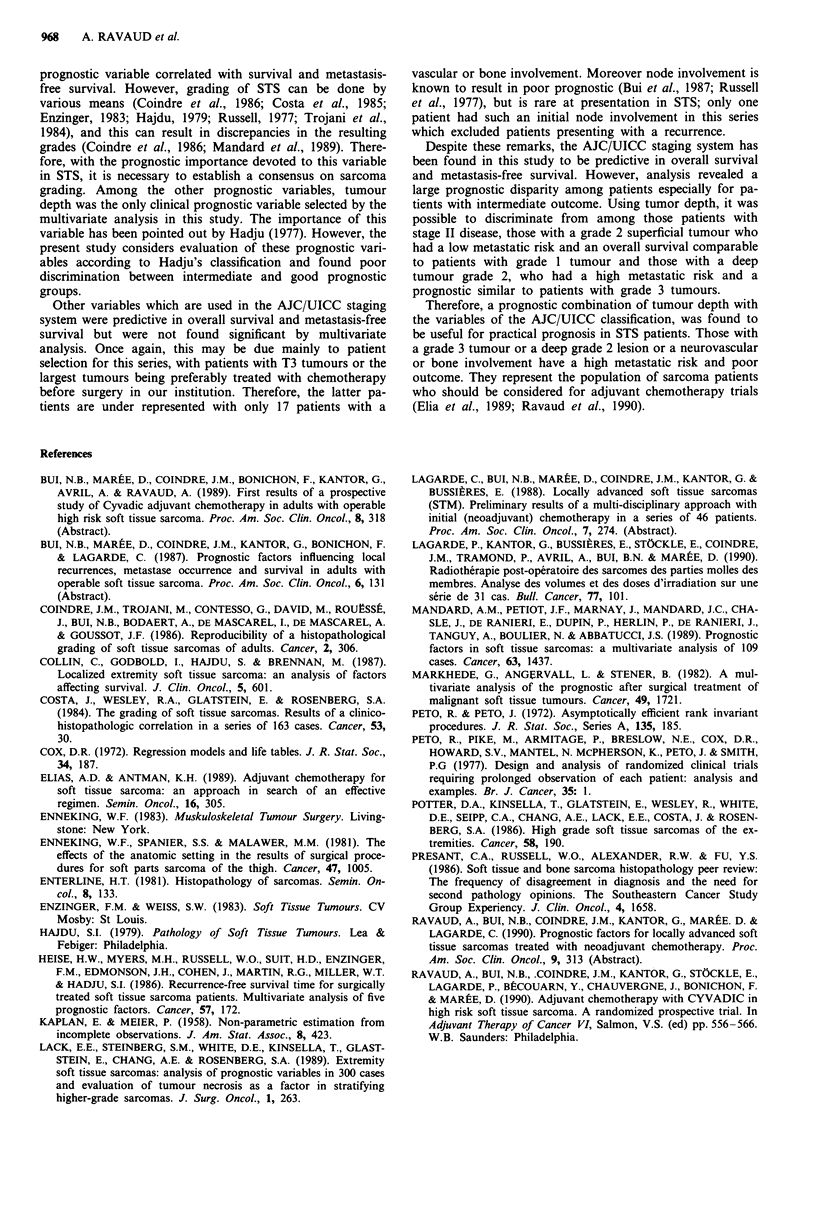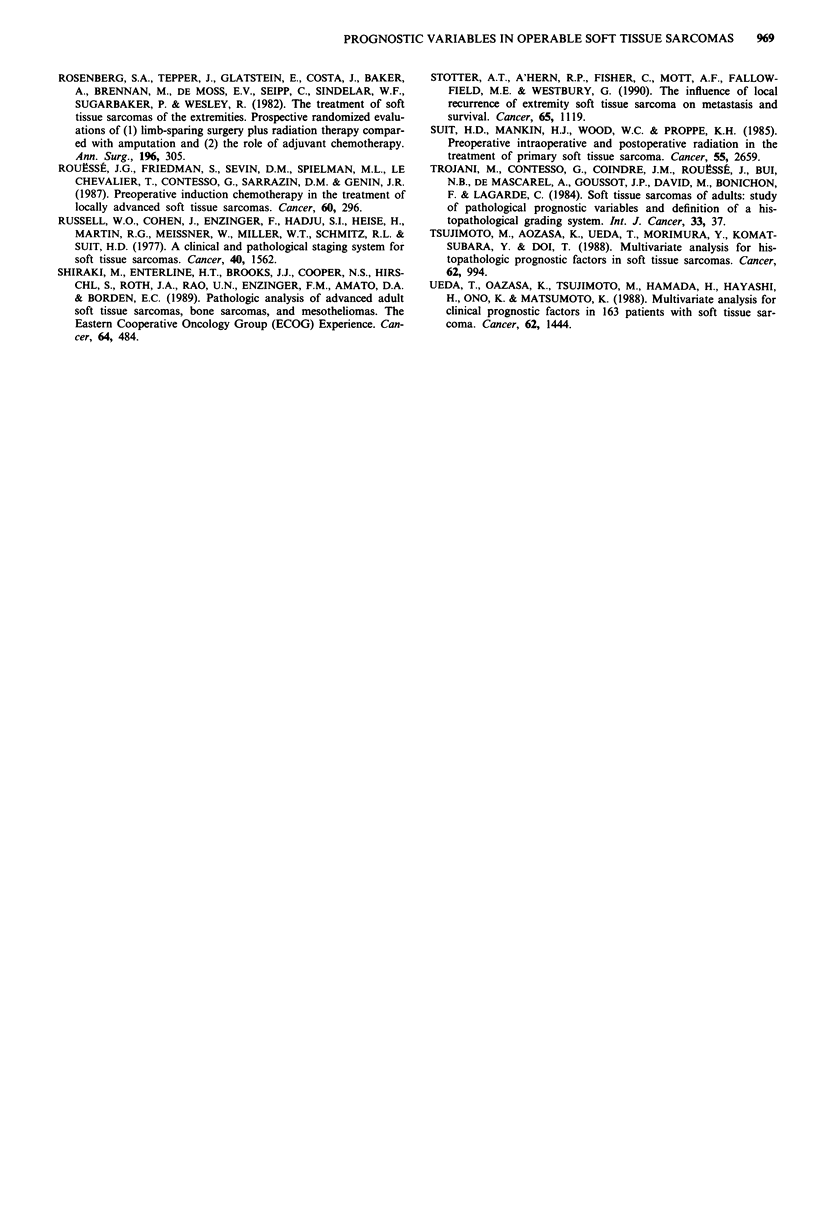# Prognostic variables for the selection of patients with operable soft tissue sarcomas to be considered in adjuvant chemotherapy trials.

**DOI:** 10.1038/bjc.1992.393

**Published:** 1992-11

**Authors:** A. Ravaud, N. B. Bui, J. M. Coindre, P. Lagarde, P. Tramond, F. Bonichon, E. Stöckle, G. Kantor, M. Trojani, J. Chauvergne

**Affiliations:** Department of Medical Oncology, Comprehensive Cancer Centre, Bordeaux, France.

## Abstract

From 1975 to 1988, 144 patients naive of treatment, with non-metastatic soft tissue sarcoma were treated at Fondation Bergonié by surgery, followed by radiotherapy and without chemotherapy. An analysis of prognostic variables was done on this population to determine patients for whom an adjuvant chemotherapy would be relevant. Prognostic variables in overall survival (OS), metastasis-free survival (MFS), disease-free and local free recurrence survivals were analysed by univariate and multivariate analysis. In multivariate analysis using Cox's model, only tumour depth and tumour grade were significant with the MFS end point, while tumour depth, tumour grade and tumour site were significant when considering OS. A predictive stratification for patients is proposed: a favourable prognostic group with grade 1 tumour or superficial, grade 2 tumour (5-year OS: 97.8%; 5-year MFS: 100%); an intermediate prognostic group with deep, grade 2 tumour or superficial, grade 3 tumour (5-year OS: 58.8%; 5-year MFS: 48.1%); and finally a poor prognostic group with deep, grade 3 tumour (5-year OS: 31.7%; 5-year MFS: 34.1%). Patients in the intermediate and poor prognostic groups who present a high metastatic risk are to be considered for adjuvant chemotherapy trials.


Br. J. Cancer (1992), 66, 961–969 © Macmillan Press Ltd., 1992

### Prognostic variables for the selection of patients with operable soft tissue sarcomas to be considered in adjuvant chemotherapy trials

A. Ravaud[1], N.B. Bui[1], J.M., Coindre[2], P. Lagarde[3], P. Tramond[4], F. Bonichon[5], E. Stöckle[4], G. Kantor[3], M. Trojani[2], J. Chauvergne[1] & D. Marée[4]

*Departments of [1]Medical Oncology, [2]Pathology, [3]Radiotherapy, [4]Surgery, [5]Biostatistics, Fondation Bergonié, Comprehensive Cancer Centre, Bordeaux, France.*

**Summary** From 1975 to 1988, 144 patients naive of treatment, with non-metastatic soft tissue sarcoma were treated at Fondation Bergonié by surgery, followed by radiotherapy and without chemotherapy. An analysis of prognostic variables was done on this population to determine patients for whom an adjuvant chemotherapy would be relevant. Prognostic variables in overall survival (OS), metastasis-free survival (MFS), disease-free and local free recurrence survivals were analysed by univariate and multivariate analysis. In multivariate analysis using Cox's model, only tumour depth and tumour grade were significant with the MFS end point, while tumour depth, tumour grade and tumour site were significant when considering OS. A predictive stratification for patients is proposed: a favourable prognostic group with grade 1 tumour or superficial, grade 2 tumour (5-year OS: 97.8%; 5-year MFS: 100%); an intermediate prognostic group with deep, grade 2 tumour or superficial, grade 3 tumour (5-year OS: 58.8%; 5-year MFS: 48.1%); and finally a poor prognostic group with deep, grade 3 tumour (5-year OS: 31.7%; 5-year MFS: 34.1%). Patients in the intermediate and poor prognostic groups who present a high metastatic risk are to be considered for adjuvant chemotherapy trials.

Soft tissue sarcomas (STS) are rare tumours, as they account for 0.7% of all malignant disease in adults. They represent an heterogeneous group of tumours which are histologically classified by their supposed histogenesis. At present, more than 60 types and subtypes are recognised (Enterline, 1981; Enzinger, 1983; Hadju, 1979).

The main advances achieved in the management of STS have been in the field of local control, which can now be obtained in approximately 80% of cases, despite important remaining problems such as the treatment of particular locations – such as the head, neck and retroperitoneum – the respective value of radical surgery versus less than radical surgery plus radiotherapy (Enneking *et al.*, 1981; Enneking 1983; Rosenberg *et al.*, 1982), optimal radiation therapy techniques (Lagarde *et al.*, 1990; Suit *et al.*, 1985), or the role of a preoperative treatment (Lagarde *et al.*, 1988; Rouëssé *et al.*, 1987). Therefore, one can consider that survival of patients with STS is mainly related to the metastatic risk of their disease. However, this metastatic evolution is widely variable from one tumour to another even within a same tumour type.

Many prognostic classifications of STS have been developed according to different evaluation of prognostic variables (Enzinger, 1983; Hajdu, 1979; Russell *et al.*, 1977). The AJC system is the most widely used, but was established by a retrospective study, and whether this system is predictive in contemporary patients remains questionable (Presant *et al.*, 1986; Russell *et al.*, 1977). Moreover, the predictive value of this classification for metastatic recurrences in the particular group of operable patients is unclear. Therefore, its suitability in selecting poor risk patients to whom adjuvant chemotherapy could be given has not been assessed.

This study was undertaken in a series of patients referred for their first treatment to one institution and managed with an homogeneous strategy for local treatment and without adjuvant chemotherapy. Only operable patients were considered to determine the poor prognostic group to be eventually selected for adjuvant treatment (Bui *et al.*, 1989; Ravaud *et al.*, 1990).

Correspondence: A. Ravaud, Foundation Bergonié, Comprehensive Cancer Centre, 180, rue de Saint-Genès, 33076 Bordeaux, France.
Received 12 August 1991; and in revised form 22 May 1992.

### Patients and methods

### *Patient selection*

From 1975 to 1988, 381 patients were referred to Fondation Bergonié (FB) for STS proven by a systematic histological review. Visceral sarcomas were not considered here. Forty-one presented with overt metastatic disease, 123 had a local recurrence after a previous treatment outside FB. The remaining 217 patients were referred for a non-metastatic and until then untreated primary. In 32 patients, a preoperative chemotherapy was performed. Moreover, 44 patients received adjuvant chemotherapy after resection of their primary; these patients were excluded. The remaining 141 patients were considered for this study because they were those for whom an adjuvant chemotherapy could prove effective, as they were: (1) naive of treatment, (2) primarily treated by surgery, (3) treated and followed at a single institution, (4) without adjuvant or neoadjuvant chemotherapy.

### *Patient characteristics*

All patients were free of metastatic disease at initial work-up which included lung tomograms before 1978, and pulmonary CAT scan after 1978. The following data were analysed from each chart: (1) patient characteristics including sex and age; (2) tumour characteristics including site, size, depth, invasion of neurovascular structures or bone, histopathology, grade, node involvement; (3) treatment characteristics including surgical procedure and results of surgery; (4) evolution including date of first local and first metastases, and status at last follow-up visit.

The histologic slides of all patients entered were reviewed at our institution by one pathologist. Immunohistochemistry was extensively used for confirmation of the diagnosis of sarcoma or for tumour typing. Tumour grade was evaluated according to the system of the French Federation of Cancer Centers (FNCLCC) based on scores obtained in the evaluation of tumour differentiation, necrosis score and mitotic count (Trojani *et al.*, 1984; Coindre *et al.*, 1986). For statistical analysis evaluating Hadju's classification, grade was defined as low with FNCLCC total grading scores from 2 to 4 and as high with FNCLCC total grading scores from 5 to 8. Tumours from all sites were considered providing a tumour excision was done primarily. Extremity tumours were subsequently divided into proximal or distal tumours at analysis. Tumours of trunk wall and those from deeper locations, such as the retroperitoneum, pelvis or mediastinum were analysed separately. Tumour size was defined as the largest tumour diameter measured either on CAT scan or on the excised specimen. A tumour situated beneath or involving the superficial fascia was considered as deep, and a tumour above the fascia as superficial. According to these characteristics, the tumours were classified according to both AJC/UICC and Hajdu's classifications.

*Patients*

Of the 141 patients, there were 77 males and 64 females, aged from 16 to 87 with a median of 50.3 years. The primary tumours were located in head and neck in 14 patients, proximal extremities in 52, distal extremities in 29, mediastinum, retroperitoneum or pelvis in 22, trunk wall including limb girdles in 24. Tumour size was less than 5 cm in only 47 patients and equal to or larger than 5 cm in 88. Forty tumours were superficial and 99 were deep. Gross or microscopic invasion of neurovascular structures or bone was present in 17 patients. Histologic types are listed in Table I with malignant fibrous histiocytoma and liposarcoma predominating. There were 30 grade 1 tumours, 67 grade 2 and 43 grade 3. Histologically confirmed involvement of regional nodes was present in only one patient. According to TNM staging, 43 patients were classified as T1, 78 as T2, 17 as T3. According to AJC/UICC staging, 29 patients were classified as stage I, 56 as stage II, 37 as stage III and 17 as stage IVa. According to Hajdu's system, 15 patients were classified as stage 0, 21 patients as stage I, 52 as stage II, 44 as stage III.

*Local treatment*

Surgery was the first treatment in all patients. The policy was to drastically limit amputations to patients with a major osseous or vasculonervous involvement considered inaccessible to 'en-bloc' resection. Compartmental surgery was not used. Wide resection, consisting of an excision of the tumour and the biopsy scar with a margin of a few centimeters of macroscopically normal tissue, was used whenever possible. Marginal resection was defined as an operation in which this margin of normal tissue could not be obtained at every point on the tumour periphery, owing either to tumour location or to the need to preserve a major vasculonervous axis or bone, adherent to the tumour but not macroscopically involved. Regional lymph node dissection was performed only in patients with an involvement proven by cytologic examination of a suspect node. For patients referred after tumour removal elsewhere, decision for reexcision was considered for each case, after clinical and radiological reevaluation of the local status, and after discussion with patients and referring physicians. However, reexcision was systematic in patients addressed 30 days or more after the initial surgery.

Consequently, the surgical procedures were amputation for only five patients, marginal resection in 64, and wide resection in 72. For seven patients, macroscopic tumour residues were left.

**Table I** Histologic types and grades

| | *I* | *II* | *III* | *Unknown* | *Total (%)* |
|---|---|---|---|---|---|
| Malignant fibrous histiocytoma | 7 | 18 | 9 | 0 | 34 (24.1) |
| Liposarcoma | 8 | 11 | 3 | 0 | 22 (15.6) |
| Leiomyosarcoma | 8 | 9 | 2 | 0 | 19 (13.5) |
| Neurosarcoma | 5 | 10 | 3 | 0 | 18 (12.8) |
| Undifferentiated sarcoma | 0 | 3 | 13 | 0 | 16 (11.3) |
| Synovial sarcoma | 0 | 10 | 3 | 0 | 13 (9.2) |
| Fibrosarcoma | 1 | 4 | 4 | 1 | 10 (7.1) |
| Angiosarcoma | 1 | 1 | 1 | 0 | 3 (2.1) |
| Rhabdomyosarcoma | 0 | 0 | 2 | 0 | 2 (1.4) |
| Extraskeletal Osteosarcoma | 0 | 0 | 2 | 0 | 2 (1.4) |
| Neuroepithelioma | 0 | 0 | 1 | 0 | 1 (0.7) |
| Clear cell sarcoma | 0 | 1 | 0 | 0 | 1 (0.7) |

Post-operative radiation therapy was not given in 31 patients: five because an amputation was performed, 18 who had wide resection of a superficial tumour and eight who had retroperitoneal tumours for which adequate post-operative irradiation was considered impossible. The target volume was planned according to pre-operative clinical examination and CT scans, surgical conditions and histopathological results. For each case, this included the tumour bed and all the tissues handled during surgery, such as scars or drain courses. The volume considered was an anatomical compartment whenever possible. In the other cases, the usual margins around the tumour-bed were 5 to 7 cm on all sides. Minimal margins could be reduced to 3 to 5 cm for particular locations such as abdominal or retroperitoneal tumours. On the other hand, the margins were enlarged for tumour patterns with a known risk of microscopic extension. Dose depended on the location of the tumour. Thus, for limb tumours and parietal lesions of the head and neck or trunk, doses were 50 Gy in 25 fractions for 5 weeks in a volume described above, with a boost of 10 Gy to 15 Gy restricted to the tumour bed. Split courses were not performed. The total dose to the final volume was 60 Gy to 65 Gy. For retroperitoneal or deep-seated abdominal tumours, the dose was reduced to 40 Gy in 25 fractions over 5 weeks with a 10 Gy boost to the tumour bed. Technical aspects of radiation treatment were discussed for each case. High energy X-rays (18 MV and 25 MV), $Co^{60}$ photon and electrons were available. Opposing fields were treated at each fraction. Simulation, portal films, and CT scan reconstitution were performed. Dose was expressed at the ICRU point, but isodose distribution curves were performed to avoid underdosage of the target volume.

**Figure 1** Overall survival and disease-free survival – all patients.

**Figure 2** Metastasis-free survival and local recurrence-free survival – all patients.

**Table II** Univariate analysis for prognostic factors in overall survival, disease-free survival, metastasis-free survival and local recurrence-free survival

| *Factors* | | *Number of patients* | | *Overall 5-year survival rate (%)* | *P value* | *Disease-free 5-year survival rate (%)* | *P value* | *Metastasis-free 5-year survival rate (%)* | *P value* | *Local recurrence-free 5-year survival rate (%)* | *P value* |
|---|---|---|---|---|---|---|---|---|---|---|---|
| *Age* (yr) | ⩽50 | 67 | | 57.7 | 0.2 | 48.6 | 0.4 | 58 | 0.2 | 69.1 | 0.2 |
| | >50 | 72 | | 73.8 | | 53 | | 67.7 | | 75.9 | |
| *Sex* | male | 77 | | 61.8 | 0.8 | 55.4 | 0.1 | 59.5 | 0.9 | 80.4 | 0.06 |
| | female | 64 | | 68.7 | | 44.4 | | 64.2 | | 63.6 | |
| *Location* | | | | | | | | | | | |
| extremities | | 81 | | 72.7 | 0.05 | 50.9 | 0.5 | 62.9 | 0.9 | 74.2 | 0.2 |
| trunk wall | | 24 | | | | | | | | | |
| head, neck | non-limb | 14 | 60 | 55.4 | | 52.2 | | 65.1 | | 72.2 | |
| pelvis, retroperitoneum | | 22 | | | | | | | | | |
| extremities | proximal | 52 | | 79.2 | 0.4 | 57.7 | 0.1 | 65.2 | 0.5 | 78.6 | 0.2 |
| | distal | 29 | 81 | 55.6 | | 40.8 | | 59.9 | | 67.4 | |
| *Tumour size* | <5 cm | 47 | | 85.7 | 0.01 | 64.3 | 0.006 | 72.4 | 0.03 | 79.4 | 0.1 |
| | ⩾5 cm | 88 | | 58 | | 47.2 | | 61.6 | | 69 | |
| *Invasion of neurovascular structure or bone* | positive | 17 | | 32.6 | 0.003 | 17.2 | 0.003 | 33.3 | 0.007 | 54.3 | 0.2 |
| | negative | 123 | | 71.6 | | 57.5 | | 67.8 | | 75.5 | |
| *TNM classification* | T1 | 43 | | 86 | 0.05 | 63.7 | 0.03 | 68.8 | 0.08 | 82.1 | 0.09 |
| | T2 | 78 | | 63.9 | 0.05 | 55.2 | 0.05 | 68.3 | 0.05 | 71.8 | 0.5 |
| | T3 | 17 | | 32.6 | | 17.2 | | 33.3 | | 54.3 | |
| *Tumour depth* | superficial | 40 | | 88.7 | 0.003 | 65.4 | 0.005 | 83 | 0.0003 | 82 | 0.1 |
| | deep | 99 | | 55.3 | | 44.4 | | 53.8 | | 67.9 | |
| *Histopathology* | | | | | | | | | | | |
| malignant fibrous histiocytoma | | 34 | | 82.7 | | 57.2 | | 82.1 | | 75.6 | |
| liposarcoma | | 22 | | 64.8 | | 73.4 | | 78.8 | | 88.5 | |
| leiomyosarcoma | | 19 | | 94.1 | | 71.1 | | 75 | | 78.9 | |
| undifferentiated sarcoma | | 16 | | 35.2 | | 33.5 | | 33.5 | | 84 | |
| synovial sarcoma | | 13 | | 41 | | 26.7 | | 37.5 | | 68.7 | |
| fibrosarcoma | | 10 | | 71.1 | | 56 | | 52.5 | | 70 | |
| malignant schwannoma | | 10 | | 100 | | 53.6 | | 85.7 | | 53.6 | |
| others | | 17 | | | | | | | | | |
| *Grade (FNCLCC)* | grade 1 | 30 | | 96.4 | | 85.4 | | 100 | | 85.4 | |
| | grade 2 | 67 | | 68.2 | <0.0001 | 49.5 | 0.0003 | 62.7 | <0.0001 | 68.3 | 0.3 |
| | grade 3 | 43 | | 41.9 | | 29.9 | | 36 | | 73.1 | |
| *Macroscopic margins* | positive | 7 | | 0 | <0.0001 | 0 | <0.0001 | 33.3 | 0.04 | 0 | <0.0001 |
| | negative | 134 | | 70.6 | | 55.1 | | 65.1 | | 76.8 | |
| *AJC/UICC Classification* | stage I | 29 | | 96.3 | 0.2 | 84.8 | 0.06 | 100 | 0.003 | 84.8 | 0.9 |
| | stage II | 56 | | 73.3 | 0.004 | 58.7 | 0.01 | 67.9 | 0.02 | 71.5 | 0.4 |
| | stage III | 37 | | 48.2 | 0.6 | 34.3 | 0.4 | 40.8 | 0.7 | 74.3 | 0.7 |
| | stage IV | 17 | | 32.6 | | 17.2 | | 33.3 | | 54.3 | |
| *HAJDU Classification* | stage 0 | 15 | | 100 | 0.2 | 92.3 | 0.3 | 100 | 0.3 | 92.3 | 0.8 |
| | stage I | 21 | | 86.4 | 0.7 | 65.8 | 0.3 | 77 | 0.3 | 89.1 | 0.1 |
| | stage II | 52 | | 78.1 | 0.0006 | 60.3 | 0.0007 | 74.4 | 0.0001 | 65.5 | 0.1 |
| | stage III | 44 | | 29.9 | | 19.3 | | 34.1 | | 59 | |

*Follow-up and statistics*

Follow-up consisted in clinical examination with chest X-ray every 2 months for 2 years, then every 4 months for 3 years, then every year. Abdominal ultrasonography were also performed in patients with intraperitoneal and abdominal tumours. Relapse or metastases was documented by biopsy or unequivocal radiographic findings.

No patient was lost for follow-up. Survival, disease-free survival, and local or metastatic recurrence-free survival were computed by the Kaplan Meier method (Kaplan, 1958). The initiation of treatment was considered as the time of origin. Death whatever the cause was considered as the event for overall survival studies, recurrence (local and/or metastatic) for disease-free survival, local recurrence for local recurrence-free survival and metastatic recurrence for metastasis-free survival.

For each variable in the univariate analysis, we used either the log-rank test (Peto, 1972) when two modalities of the variable were compared, or a test for trend (Peto, 1977) when three modalities were tested. Each variable was studied for each of the following end points: local recurrence, metastatic recurrence and overall survival. The variables, with $P$ value $<0.10$ in the univariate analysis, were included in Cox's models (Cox, 1972) (program 2L of BMDP statistical software). A stepwise procedure using MPLR (maximum partial likehood ratio) made it possible to summarise the prognostic information with the most pertinent variables. If $a_i$ are the estimated coefficients for the final model and $X_i$ the value of the $i^{th}$ independent variable for the patient j, a score $S_j$ can be computed as follows:

$$S_j = a_1 X_1 + a_2 X_2 + \ldots + a_n X_n$$

This defined score makes it possible to rank each patient on a scale with an increasing risk. Score values may be delimited to determine different prognostic groups.

### Results

With a median follow-up of 57.6 months (range 11–168 months), 11 patients (7.8%) died from a cause unrelated to the treated sarcoma: cardiac or vascular disease: four; cerebral palsy: one, pulmonary infection: one; diabetes mellitus: one; suicide: one; other cancer: three (osteosarcoma, colon cancer, renal cancer); three of these patients presented a local recurrence and one a metastatic spread. These patients were excluded from the overall survival analysis but not from disease-free or metastasis-free survival evaluations. At their last follow-up visit, 90 patients were alive without evidence of disease (63.8%), six were alive with recurrence (4.3%), 34 had died from STS (24.1%); for the entire series, the actuarial 5-year overall survival rate was 66.1% (Figure 1); the disease-free 5-year survival rate was 51.4% (Figure 1); the metastasis-free 5-year survival rate was 63.2% (Figure 2) and the local recurrence-free 5-year survival rate was 73.1% (Figure 2).

### Univariate analysis (Table II)

*Age and sex* did not affect patient evolution, although there was a trend for a better local recurrence-free survival in male patients ($P = 0.06$).

**Figure 3** Overall survival in patients with superficial or deep tumours. Superficial (40 pts), deep (99 pts).

**Figure 4** Disease-free (DFS) and metastasis-free (MFS) survival – superficial or deep tumour. Superficial (40 pts), deep (99 pts).

**Figure 5** Overall survival according to grade (FNCLCC). Grade 1 (30 pts), grade 2 (67 pts), grade 3 (43 pts).

**Figure 6** Metastasis-free survival according to grade. Grade 1 (30 pts), grade 2 (67 pts), grade 3 (43 pts).

*Location* Overall survival was significantly better in patients with limb primaries compared to patients with non-limb tumours ($P = 0.05$). However, there was no difference regarding local control or metastasis-free survival. The only point which could explain a difference only in overall survival was a significantly better outcome after rescue treatment in patients with an extremity tumour who first experienced a relapse. For limb tumours, no significant difference was observed regarding a proximal or distal location.

*Tumour size* The median largest tumour diameter in this series was 8.2 cm (range 1–40 cm). Patients with tumours less than 5 cm had a significantly better overall survival ($P = 0.01$), disease-free survival ($P = 0.006$) and metastasis-free survival ($P = 0.03$) than those with a tumour of 5 cm or more. There was no significant difference in terms of local recurrence-free survival ($P = 0.14$).

*Invasion of neurovascular structures or bone* Patients with such an invasion had a significantly worse prognostic in overall survival ($P = 0.003$), disease-free survival ($P = 0.003$) and metastasis-free survival ($P = 0.007$) than those without invasion.

*T staging* Owing to differences according to tumour size and local invasion, there was a significant difference in evolution between T1, T2, T3 lesions, as defined by the AJC staging system, except for local recurrence-free survival.

*Tumour depth* Tumour depth had a significant impact on prognostic. Patients with superficial lesions had a significantly better overall survival ($P = 0.003$) (Figure 3), disease-free survival ($P = 0.005$) and metastasis-free survival ($P = 0.0003$) (Figure 4) than those with deep tumours. Tumour depth did not affect local control.

**Figure 7** Overall survival according to stage (AJC/UICC classification). Stage I (29 pts), stage II (56 pts), stage III (37 pts), stage IVa (17 pts).

**Figure 8** Metastasis-free survival according to stage (AJC/UICC classification). Stage I (29 pts), stage II (56 pts), stage III (37 pts), stage IVa (17 pts).

*Histopathology* Sixty-five per cent of the tumours could be classifed into four histopathological types: malignant fibrous histocytoma (MFH) (24.1%), liposarcoma (15.6%), leiomyosarcoma (13.5%), undifferentiated sarcoma (11.3%). Three spindle cell sarcomas were classified as undifferentiated sarcoma and were classified as grade 2 tumours. In this series, the 5-year overall survival was over 65% for patients with the following tumours: MFH (82.7%), liposarcoma (64.8%), leiomyosarcoma (94.1%), neurosarcoma (68.9%) or fibrosarcoma (71.1%). Furthermore, overall survival was lower than 50% in patients with undifferentiated sarcoma (35.2%) and synovial sarcoma (41%) (Table II). There was a similar trend for 5-year metastasis-free survival. However, the low number of patients for each tumour type group did not allow a correct statistical analysis. Moreover, distribution of tumour grade was different according to tumour type; grade 3 were more frequent in the histologic types with the poorest prognostic such as undifferentiated sarcoma or synovial sarcoma.

*Grade* Tumour grade was found to be strongly correlated with overall survival ($P < 0.0001$) (Figure 5), disease-free survival ($P = 0.0003$) and metastasis-free survival ($P < 0.001$) (Figure 6). No patient with a grade 1 lesion presented a metastatic relapse at the time of analysis.

*Macroscopic margins* A macroscopic tumour residue was left in seven patients after surgery. These patients had a

**Table III** Stepwise Cox's multivariate analysis for prognostic factors in overall survival (Mantel-Cox)

| *Step* | *Variable entered (code)* | *Log likelihood* | *Global chi-square* | *P value* |
|---|---|---|---|---|
| 0 | | – 136.741 | | |
| 1 | Grade (grade 1 = 1; grade 2 = 2; grade 3 = 3) | – 126.472 | 19.725 | 0.0000089 |
| 2 | Tumour Depth (0 = superficial; 1 = deep) | – 120.534 | 29.672 | 0.0000003 |
| 3 | Tumour site (0 = non-limb; 1 = limb) | – 118.010 | 34.562 | 0.0000001 |

No other term (tumour size, invasion of neurovascular structures or bone, macroscopic margins) passes the remove and enter limits (0.10; 0.05)

| *Variable* | *Coeff.* | *Coeff./standard error* | *Exp (coeff.)* | *P value* |
|---|---|---|---|---|
| Grade | 1.3348 | 4.5983 | 3.7994 | 0.000004 |
| Tumour depth | 1.6414 | 3.0157 | 5.1624 | 0.0025 |
| Tumour site | – 0.8296 | – 2.2459 | 0.4362 | 0.025 |

Table IV Stepwise Cox's multivariate analysis for prognostic factors in disease-free survival (Mantel-Cox)

| Step | Variable entered (code) | Log likelihood | Global chi-square | P value |
|---|---|---|---|---|
| 0 | | – 209.589 | | |
| 1 | Grade (grade 1 = 1; grade 2 = 2; grade 3 = 3) | – 201.181 | 16.5 | 0.00005 |
| 2 | Tumour size (0 = < 5 cm; 1 = ⩾ 5 cm) | – 196.696 | 24.898 | 0.000004 |

No other term (tumour location, tumour depth, invasion of neurovascular structures or bone, macroscopic margins) passes the remove and enter limits (0.10; 0.05)

| Variable | Coeff./standard error | Exp (coeff.) | P value |
|---|---|---|---|
| Grade | 4.0460 | 2.4072 | 0.00005 |
| Tumour site | 2.7321 | 2.7723 | 0.006 |

Table V Stepwise Cox's multivariate analysis for prognostic factors in metastasis-free survival (Mantel-Cox)

| Step | Variable entered (code) | Log likelihood | Global chi-square | P value |
|---|---|---|---|---|
| 0 | | 149.366 | | |
| 1 | Grade (grade 1 = 1; grade 2 = 2; grade 3 = 3) | – 137.771 | 22.155 | 0.0000025 |
| 2 | Tumour Depth (0 = superficial; 1 = deep) | – 129.393 | 36.190 | 0.00000001 |

No other term (tumour location, tumour size, invasion of neurovascular structures or bone, macroscopic margins) passes the remove and enter limits (0.10; 0.05)

| Variable | Coeff. | Coeff./standard error | Exp (coeff.) | P value |
|---|---|---|---|---|
| Grade | 1.31 | 4.5854 | 7.0413 | 0.0000046 |
| Tumour depth | 1.95 | 3.2021 | 3.7065 | 0.0013 |

Table VI Stepwise Cox's multivariate analysis for prognostic factors in local recurrence-free survival (Mantel-Cox)

| Step | Variable entered (code) | Log likelihood | Global chi-square | P value |
|---|---|---|---|---|
| 0 | | – 110.916 | | |
| 1 | Macroscopic margins (0 = negative; 1 = positive) | – 106.135 | 18.687 | 0.000015 |
| 2 | Sex (1 = male; 2 = female) | – 102.591 | 25.702 | 0.0000026 |

No other term (tumour location, tumour size, tumour depth, grade, invasion of neurovascular structures) passes the remove and enter limits (0.10; 0.05)

| Variable | Coeff./standard error | Exp (coeff.) | P value |
|---|---|---|---|
| Macroscopic margins | 3.8309 | 7.1997 | 0.00013 |
| Sex | 2.5507 | 3.0040 | 0.01 |

lower overall survival ($P < 0.0001$), disease-free survival ($P < 0.0001$) and metastasis-free survival ($P = 0.04$).

*AJC/UICC classification* Prognostic groups established according to tumour size, local tumour invasiveness, tumour grade, and node status according to the AJC/UICC classification were predictive for overall survival (Figure 7), metastasis-free survival (Figure 8) and disease-free survival. There was no significant difference between stage III and stage IVa, but this latter subgroup is poorly represented in this study owing to patient selection. The AJC/UICC appears to be more discriminating for prognostic evaluation than Hajdu's classification, which considers tumour size, grade and depth, where no significant difference appeared between stage 0, I or II. However, there was a significant difference in overall survival ($P = 0.0006$), disease-free survival ($P = 0.0007$) and metastasis-free survival ($P = 0.0001$) between stages II and III.

**Multivariate analysis (Tables III, IV, V, VI)**

The six prognostic variables (tumour site, tumour size, tumour depth, invasion of neurovascular structures or bone, grade and macroscopic margins) found to be significantly correlated with evolution in univariate analysis were selected for a Cox's stepwise multivariate analysis. This analysis was done in 131 patients with no missing data. Tumour grade, and tumour depth was the most significant predictive variables for metastasis-free survival (Table III). Furthermore, these two variables and tumour site were predictive factors for overall survival (Table IV). On the other hand, macroscopic margins and sex were significant for local recurrence (Table VI).

According to the end points considered in this study, each patient with STS could be scored for MFS and OS, considering the coefficient value of each significant variable determined by Cox's multivariate analysis (Table III, V).

**Table VII** Prognostic score in metastasis-free survival according to the stepwise Cox's multivariate analysis (Table V)

| *Tumour characteristics* | *Tumour grade grade 1 = 1; grade 2 = 2; grade 3 = 3 coefficient: 1.31* | | *Tumour depth superficial = 0; deep = 0 coefficient: 1.95* | | *Score* | *Increasing metastatic risk* |
|---|---|---|---|---|---|---|
| Grade 1 and superficial | 1 × 1.31 | + | 0 × 1.95 | = | 1.31 | |
| Grade 2 and superficial | 2 × 1.31 | + | 0 × 1.95 | = | 2.62 | |
| Grade 1 and deep | 1 × 1.31 | + | 1 × 1.95 | = | 3.26 | |
| Grade 3 and superficial | 3 × 1.31 | + | 0 × 1.95 | = | 3.93 | |
| Grade 2 and deep | 2 × 1.31 | + | 1 × 1.95 | = | 4.57 | |
| Grade 3 and deep | 3 × 1.31 | + | 1 × 1.95 | = | 5.88 | |

**Figure 9** Overall survival according to prognostic groups. Grade 1 + superficial grade 2 (49 pts), deep grade 2 + superficial grade 3 (52 pts), deep grade 3 (30 pts).

**Figure 10** Metastasis-free survival according to prognostic groups. Grade 1 + superficial grade 2 (49 pts), deep grade 2 + superficial grade 3 (52 pts), deep grade 3 (30 pts).

For example, Table VII represents the increasing metastatic risk for this series. Consequently, tumour grade and deepness were considered for a prognostic stratification and allowed classification of patients into three groups. A favorable prognostic group included patients with grade 1 tumour or superficial, grade 2 tumour and had a 5-year corrected survival rate of 97.8% and a 5-year metastasis-free survival rate of 100% (Figure 9, 10). An intermediate prognostic group with patients, with deep, grade 2 tumour or superficial, grade 3 tumour, had a 5-year overall survival rate of 58.8% and a 5-year metastasis-free survival of 48.1%. Finally, a poor prognostic group defined as patients with deep grade 3 tumour had a 5-year survival rate of 31.7% and a 5-year metastasis-free survival of 34.1%.

### Discussion

Whether adjuvant chemotherapy is of benefit or not in patients with operable STS remains a matter of controversy (Elias, 1989; Ravaud *et al.*, 1990). Among the reasons which can be called upon to explain the discrepancies in results from the different clinical trials performed, there is the difference in the selection of poor risk patients, owing to differences in evaluation of prognostic variables (Coindre *et al.*, 1986; Collin *et al.*, 1987; Costa *et al.*, 1984; Enzinger, 1983; Hajdu, 1979; Heise *et al.*, 1986; Lack *et al.*, 1989; Mandard *et al.*, 1989; Markhede *et al.*, 1982; Potter *et al.*, 1986; Presant *et al.*, 1986; Russel, 1977; Shiraki *et al.*, 1989; Stotter *et al.*, 1990; Trojani *et al.*, 1984; Tsujimoto, 1988; Ueda *et al.*, 1988). This study confirms that the evolution of patients with an operable STS depends mainly on the eventual advent of metastatic disease: of the 34 patients who died from sarcoma, death was due to a metastatic spread in 30/34 patients (88.3%) and only 4/34 patients (11.3%) died of local tumour evolution. Therefore, the main end point to define high-risk patients must be metastasis-free survival rather than overall survival or disease-free survival. In this study, the prognostic variables which confer the highest relative risk for metastatic disease were tumour grade and tumour depth. These variables have also been found of prognostic importance in previous studies, although dealing with other patient selection criteria (Trojani *et al.*, 1984).

In this series, tumour location (limb tumours versus non-limb tumours) had an impact only on overall survival. The only point which could explain the difference in overall survival, while no significant difference in local recurrence and metastatic rate was found, was a better outcome following rescue treatments in patients with an extremity tumour who experienced a local relapse. That may also be due in part to the patient selection modalities used to allow a primarily conservative surgery. Patients who did not fit those selection criteria were included in another treatment program with systematic neoadjuvant chemotherapy; among those patients, 62% had non-limb tumours (Ravaud *et al.*, 1990).

Previous studies have pointed out other significant prognostic variables such as tumour size, invasion of adjacent neurovascular structures or bone, histopathological types and surgical margins.

In this series as in others (Collin *et al.*, 1987; Heise *et al.*, 1986; Lack *et al.*, 1989; Markhede *et al.*, 1982; Potter *et al.*, 1986; Trojani *et al.*, 1984) tumour histopathological type was difficult to correlate with prognostic, although better prognostic was associated with certain types, while others like synovial sarcoma or undifferentiated sarcoma were more ominous. However, the rarity of sarcomas and the multiplicity of tumour types and subtypes lead to considerable difficulty in pathological classification (Coindre *et al.*, 1986; Presant *et al.*, 1986; Shiraki *et al.*, 1989). Moreover, as results of tumour histopathological classification are highly variable from one pathologist to another, a prognostic evaluation based on tumour type appears hazardous. In fact, histopathological type has already been reported to be less predictive of the evolution of sarcoma than tumour grade (Markhede *et al.*, 1982).

In this series as in others (Coindre *et al.*, 1986; Collin *et al.*, 1987; Costa *et al.*, 1984; Enzinger, 1983; Hajdu, 1979; Heise *et al.*, 1986; Lack *et al.*, 1989; Mandard *et al.*, 1989; Markhede *et al.*, 1982; Potter *et al.*, 1986; Presant *et al.*, 1986; Russell, 1977; Shiraki *et al.*, 1989; Stotter *et al.*, 1990; Trojani *et al.*, 1984; Tsujimoto, 1988; Ueda *et al.*, 1988), tumour grade was found to be the most important single prognostic variable correlated with survival and metastasis-free survival. However, grading of STS can be done by various means (Coindre *et al.*, 1986; Costa *et al.*, 1985; Enzinger, 1983; Hajdu, 1979; Russell, 1977; Trojani *et al.*, 1984), and this can result in discrepancies in the resulting grades (Coindre *et al.*, 1986; Mandard *et al.*, 1989). Therefore, with the prognostic importance devoted to this variable in STS, it is necessary to establish a consensus on sarcoma grading. Among the other prognostic variables, tumour depth was the only clinical prognostic variable selected by the multivariate analysis in this study. The importance of this variable has been pointed out by Hadju (1977). However, the present study considers evaluation of these prognostic variables according to Hadju's classification and found poor discrimination between intermediate and good prognostic groups.

Other variables which are used in the AJC/UICC staging system were predictive in overall survival and metastasis-free survival but were not found significant by multivariate analysis. Once again, this may be due mainly to patient selection for this series, with patients with T3 tumours or the largest tumours being preferably treated with chemotherapy before surgery in our institution. Therefore, the latter patients are under represented with only 17 patients with a vascular or bone involvement. Moreover node involvement is known to result in poor prognostic (Bui *et al.*, 1987; Russell *et al.*, 1977), but is rare at presentation in STS; only one patient had such an initial node involvement in this series which excluded patients presenting with a recurrence.

Despite these remarks, the AJC/UICC staging system has been found in this study to be predictive in overall survival and metastasis-free survival. However, analysis revealed a large prognostic disparity among patients especially for patients with intermediate outcome. Using tumor depth, it was possible to discriminate from among those patients with stage II disease, those with a grade 2 superficial tumour who had a low metastatic risk and an overall survival comparable to patients with grade 1 tumour and those with a deep tumour grade 2, who had a high metastatic risk and a prognostic similar to patients with grade 3 tumours.

Therefore, a prognostic combination of tumour depth with the variables of the AJC/UICC classification, was found to be useful for practical prognosis in STS patients. Those with a grade 3 tumour or a deep grade 2 lesion or a neurovascular or bone involvement have a high metastatic risk and poor outcome. They represent the population of sarcoma patients who should be considered for adjuvant chemotherapy trials (Elia *et al.*, 1989; Ravaud *et al.*, 1990).

### References

BUI, N.B., MARÉE, D., COINDRE, J.M., BONICHON, F., KANTOR, G., AVRIL, A. & RAVAUD, A. (1989). First results of a prospective study of Cyvadic adjuvant chemotherapy in adults with operable high risk soft tissue sarcoma. *Proc. Am. Soc. Clin. Oncol.*, **8**, 318 (Abstract).

BUI, N.B., MARÉE, D., COINDRE, J.M., KANTOR, G., BONICHON, F. & LAGARDE, C. (1987). Prognostic factors influencing local recurrences, metastase occurrence and survival in adults with operable soft tissue sarcoma. *Proc. Am. Soc. Clin. Oncol.*, **6**, 131 (Abstract).

COINDRE, J.M., TROJANI, M., CONTESSO, G., DAVID, M., ROUËSSÉ, J., BUI, N.B., BODAERT, A., DE MASCAREL, I., DE MASCAREL, A. & GOUSSOT, J.F. (1986). Reproducibility of a histopathological grading of soft tissue sarcomas of adults. *Cancer*, **2**, 306.

COLLIN, C., GODBOLD, I., HAJDU, S. & BRENNAN, M. (1987). Localized extremity soft tissue sarcoma: an analysis of factors affecting survival. *J. Clin. Oncol.*, **5**, 601.

COSTA, J., WESLEY, R.A., GLATSTEIN, E. & ROSENBERG, S.A. (1984). The grading of soft tissue sarcomas. Results of a clinico-histopathologic correlation in a series of 163 cases. *Cancer*, **53**, 30.

COX, D.R. (1972). Regression models and life tables. *J. R. Stat. Soc.*, **34**, 187.

ELIAS, A.D. & ANTMAN, K.H. (1989). Adjuvant chemotherapy for soft tissue sarcoma: an approach in search of an effective regimen. *Semin. Oncol.*, **16**, 305.

ENNEKING, W.F. (1983). *Muskuloskeletal Tumour Surgery*. Livingstone: New York.

ENNEKING, W.F., SPANIER, S.S. & MALAWER, M.M. (1981). The effects of the anatomic setting in the results of surgical procedures for soft parts sarcoma of the thigh. *Cancer*, **47**, 1005.

ENTERLINE, H.T. (1981). Histopathology of sarcomas. *Semin. Oncol.*, **8**, 133.

ENZINGER, F.M. & WEISS, S.W. (1983). *Soft Tissue Tumours*. CV Mosby: St Louis.

HAJDU, S.I. (1979). *Pathology of Soft Tissue Tumours*. Lea & Febiger: Philadelphia.

HEISE, H.W., MYERS, M.H., RUSSELL, W.O., SUIT, H.D., ENZINGER, F.M., EDMONSON, J.H., COHEN, J., MARTIN, R.G., MILLER, W.T. & HADJU, S.I. (1986). Recurrence-free survival time for surgically treated soft tissue sarcoma patients. Multivariate analysis of five prognostic factors. *Cancer*, **57**, 172.

KAPLAN, E. & MEIER, P. (1958). Non-parametric estimation from incomplete observations. *J. Am. Stat. Assoc.*, **8**, 423.

LACK, E.E., STEINBERG, S.M., WHITE, D.E., KINSELLA, T., GLASTSTEIN, E., CHANG, A.E. & ROSENBERG, S.A. (1989). Extremity soft tissue sarcomas: analysis of prognostic variables in 300 cases and evaluation of tumour necrosis as a factor in stratifying higher-grade sarcomas. *J. Surg. Oncol.*, **1**, 263.

LAGARDE, C., BUI, N.B., MARÉE, D., COINDRE, J.M., KANTOR, G. & BUSSIÈRES, E. (1988). Locally advanced soft tissue sarcomas (STM). Preliminary results of a multi-disciplinary approach with initial (neoadjuvant) chemotherapy in a series of 46 patients. *Proc. Am. Soc. Clin. Oncol.*, **7**, 274. (Abstract).

LAGARDE, P., KANTOR, G., BUSSIÈRES, E., STÖCKLE, E., COINDRE, J.M., TRAMOND, P., AVRIL, A., BUI, B.N. & MARÉE, D. (1990). Radiothérapie post-opératoire des sarcomes des parties molles des membres. Analyse des volumes et des doses d'irradiation sur une série de 31 cas. *Bull. Cancer*, **77**, 101.

MANDARD, A.M., PETIOT, J.F., MARNAY, J., MANDARD, J.C., CHASLE, J., DE RANIERI, E., DUPIN, P., HERLIN, P., DE RANIERI, J., TANGUY, A., BOULIER, N. & ABBATUCCI, J.S. (1989). Prognostic factors in soft tissue sarcomas: a multivariate analysis of 109 cases. *Cancer*, **63**, 1437.

MARKHEDE, G., ANGERVALL, L. & STENER, B. (1982). A multivariate analysis of the prognostic after surgical treatment of malignant soft tissue tumours. *Cancer*, **49**, 1721.

PETO, R. & PETO, J. (1972). Asymptotically efficient rank invariant procedures. *J. R. Stat. Soc.*, Series A, **135**, 185.

PETO, R., PIKE, M., ARMITAGE, P., BRESLOW, N.E., COX, D.R., HOWARD, S.V., MANTEL, N. MCPHERSON, K., PETO, J. & SMITH, P.G (1977). Design and analysis of randomized clinical trials requiring prolonged observation of each patient: analysis and examples. *Br. J. Cancer*, **35**: 1.

POTTER, D.A., KINSELLA, T., GLATSTEIN, E., WESLEY, R., WHITE, D.E., SEIPP, C.A., CHANG, A.E., LACK, E.E., COSTA, J. & ROSENBERG, S.A. (1986). High grade soft tissue sarcomas of the extremities. *Cancer*, **58**, 190.

PRESANT, C.A., RUSSELL, W.O., ALEXANDER, R.W. & FU, Y.S. (1986). Soft tissue and bone sarcoma histopathology peer review: The frequency of disagreement in diagnosis and the need for second pathology opinions. The Southeastern Cancer Study Group Experiency. *J. Clin. Oncol.*, **4**, 1658.

RAVAUD, A., BUI, N.B., COINDRE, J.M., KANTOR, G., MARÉE. D. & LAGARDE, C. (1990). Prognostic factors for locally advanced soft tissue sarcomas treated with neoadjuvant chemotherapy. *Proc. Am. Soc. Clin. Oncol.*, **9**, 313 (Abstract).

RAVAUD, A., BUI, N.B., .COINDRE, J.M., KANTOR, G., STÖCKLE, E., LAGARDE, P., BÉCOUARN, Y., CHAUVERGNE, J., BONICHON, F. & MARÉE, D. (1990). Adjuvant chemotherapy with CYVADIC in high risk soft tissue sarcoma. A randomized prospective trial. In *Adjuvant Therapy of Cancer VI*, Salmon, V.S. (ed) pp. 556–566. W.B. Saunders: Philadelphia.

ROSENBERG, S.A., TEPPER, J., GLATSTEIN, E., COSTA, J., BAKER, A., BRENNAN, M., DE MOSS, E.V., SEIPP, C., SINDELAR, W.F., SUGARBAKER, P. & WESLEY, R. (1982). The treatment of soft tissue sarcomas of the extremities. Prospective randomized evaluations of (1) limb-sparing surgery plus radiation therapy compared with amputation and (2) the role of adjuvant chemotherapy. *Ann. Surg.*, **196**, 305.

ROUËSSÉ, J.G., FRIEDMAN, S., SEVIN, D.M., SPIELMAN, M.L., LE CHEVALIER, T., CONTESSO, G., SARRAZIN, D.M. & GENIN, J.R. (1987). Preoperative induction chemotherapy in the treatment of locally advanced soft tissue sarcomas. *Cancer*, **60**, 296.

RUSSELL, W.O., COHEN, J., ENZINGER, F., HADJU, S.I., HEISE, H., MARTIN, R.G., MEISSNER, W., MILLER, W.T., SCHMITZ, R.L. & SUIT, H.D. (1977). A clinical and pathological staging system for soft tissue sarcomas. *Cancer*, **40**, 1562.

SHIRAKI, M., ENTERLINE, H.T., BROOKS, J.J., COOPER, N.S., HIRSCHL, S., ROTH, J.A., RAO, U.N., ENZINGER, F.M., AMATO, D.A. & BORDEN, E.C. (1989). Pathologic analysis of advanced adult soft tissue sarcomas, bone sarcomas, and mesotheliomas. The Eastern Cooperative Oncology Group (ECOG) Experience. *Cancer*, **64**, 484.

STOTTER, A.T., A'HERN, R.P., FISHER, C., MOTT, A.F., FALLOWFIELD, M.E. & WESTBURY, G. (1990). The influence of local recurrence of extremity soft tissue sarcoma on metastasis and survival. *Cancer*, **65**, 1119.

SUIT, H.D., MANKIN, H.J., WOOD, W.C. & PROPPE, K.H. (1985). Preoperative intraoperative and postoperative radiation in the treatment of primary soft tissue sarcoma. *Cancer*, **55**, 2659.

TROJANI, M., CONTESSO, G., COINDRE, J.M., ROUËSSÉ, J., BUI, N.B., DE MASCAREL, A., GOUSSOT, J.P., DAVID, M., BONICHON, F. & LAGARDE, C. (1984). Soft tissue sarcomas of adults: study of pathological prognostic variables and definition of a histopathological grading system. *Int. J. Cancer*, **33**, 37.

TSUJIMOTO, M., AOZASA, K., UEDA, T., MORIMURA, Y., KOMATSUBARA, Y. & DOI, T. (1988). Multivariate analysis for histopathologic prognostic factors in soft tissue sarcomas. *Cancer*, **62**, 994.

UEDA, T., OAZASA, K., TSUJIMOTO, M., HAMADA, H., HAYASHI, H., ONO, K. & MATSUMOTO, K. (1988). Multivariate analysis for clinical prognostic factors in 163 patients with soft tissue sarcoma. *Cancer*, **62**, 1444.